# Social Ecological Influences on HPV Vaccination Among Cape Verdean Immigrants in the U. S.: A Qualitative Study

**DOI:** 10.3390/vaccines13070713

**Published:** 2025-06-30

**Authors:** Ana Cristina Lindsay, Celestina V. Antunes, Aysha G. Pires, Monica Pereira, Denise L. Nogueira

**Affiliations:** 1 Department of Urban Public Health, Donna M & Robert J Manning College of Nursing & Health Sciences, University of Massachusetts Boston, Boston, MA 02125, USA; 2 Department of Exercise and Health Sciences, Donna M & Robert J Manning College of Nursing & Health Sciences, University of Massachusetts Boston, Boston, MA 02125, USA; celestina.antunes001@umb.edu (C.V.A.); aysha.pires001@umb.edu (A.G.P.); monica.pereira@umb.edu (M.P.); 3 Department of Nursing, Faculty Luciano Feijão, Sobral 62050-215, CE, Brazil; deniseln2009@hotmail.com

**Keywords:** human papillomavirus vaccination, HPV, qualitative research, Cape Verdean, African immigrants, social ecological model

## Abstract

**Background:** Human papillomavirus (HPV) is the most common sexually transmitted infection in the United States (U.S.) and a major contributor to several cancers, including cervical, anal, penile, and oropharyngeal cancers. Although a safe and effective vaccine is available, HPV vaccination rates remain suboptimal, particularly among racial, ethnic, and immigrant minority groups. This study explored multiple factors, such as cultural, social, and structural influences, influencing HPV vaccine decision-making among Cape Verdean immigrant parents in the U.S., a population currently underrepresented in HPV research. **Methods:** Qualitative study using individual, in-depth interviews with Cape Verdean immigrant parents of children aged 11 to 17 years living in the U.S. Interviews were transcribed verbatim and analyzed thematically using the social ecological model (SEM) to identify barriers and facilitators at the intrapersonal, interpersonal, organizational, community, and policy levels. **Results:** Forty-five Cape Verdean parents (27 mothers, 18 fathers) participated. Fathers were significantly older than mothers (50.0 vs. 41.1 years, *p* = 0.05). Most were married or partnered (60%), had at least a high school education (84.4%), and reported annual household incomes of US$50,000 or more (66.7%), with no significant gender differences. Nearly all spoke Creole at home (95.6%). Fathers had lower acculturation than mothers (*p* = 0.05), reflecting less adaptation to U.S. norms and language use. Most parents had limited knowledge of HPV and the vaccine, with gendered beliefs and misconceptions about risk. Only seven mothers (25.9%) reported receiving a provider recommendation; all indicated that their children had initiated vaccination (1 dose or more). Mothers were the primary decision-makers, though joint decision-making was common. Trust in providers was high, but poor communication and the lack of culturally and linguistically appropriate materials limited informed decision-making. Stigma, misinformation, and cultural taboos restricted open dialogue. Trusted sources of information included schools, churches, and Cape Verdean organizations. While parents valued the U.S. healthcare system, they noted gaps in public health messaging and provider engagement. **Conclusions:** Findings revealed that HPV vaccine uptake and hesitancy among Cape Verdean immigrant parents in the U.S. were influenced by individual beliefs, family dynamics, healthcare provider interactions, cultural norms, and structural barriers. These findings highlight the need for multilevel strategies such as culturally tailored education, community engagement, and improved provider communication to support informed vaccination decisions in this population.

## 1. Introduction

Human papillomavirus (HPV) is the most common sexually transmitted infection in the United States (U.S.) and a major cause of several cancers, including cervical, anal, penile, and oropharyngeal cancers [1,2]. Despite the availability of a safe and effective vaccine, HPV vaccination rates remain low, particularly among racial and ethnic minority groups, including immigrants [3,4]. 

The Centers for Disease Control and Prevention (CDC) recommends routine HPV vaccination for children beginning at age 11 or 12, with the option to start as early as age 9 [5]. Nonetheless, disparities in vaccine uptake, both in the U.S. and globally, underscore the need to better understand the multilevel factors influencing vaccination decisions among underserved populations, especially given HPV’s strong association with multiple types of cancer [6,7,8,9].

Cape Verdean immigrants, an Afro-Portuguese group, represent a distinct and underrepresented population within the broader African diaspora in the U.S. [10]. Primarily residing in Northeastern states like Massachusetts, Rhode Island, and Connecticut, they maintain strong linguistic, cultural, and familial ties to Cape Verde, which can influence health behaviors [10,11]. Like other immigrant groups, they face systemic barriers such as limited healthcare access, language challenges, and misinformation, all of which may hinder HPV vaccination uptake [12,13,14]. Cultural beliefs and community dynamics further influence vaccination decisions [15,16,17,18,19]. Although these challenges persist, public health research has given limited attention to specific immigrant populations such as Cape Verdeans [20].

Although Cape Verdeans have a long-established presence in the U.S., little is known about their parental perspectives and decision-making regarding HPV vaccination. This gap limits the development of culturally appropriate interventions aimed at reducing health disparities in this underserved population.

In Cape Verde, cervical cancer is the third most common cancer among women and the leading cause of cancer-related death [21]. In April 2021, the Cape Verdean government introduced the HPV vaccine into its national immunization program to improve awareness and coverage among adolescent girls [21]. Because this program is relatively new, Cape Verdean immigrants in the U.S., many of whom retain strong ties to their home country, may have differing levels of exposure to HPV-related education and prevention efforts [10,11]. These transnational health experiences are important when examining HPV vaccine attitudes among Cape Verdean parents in the U.S., as they can shape beliefs, risk perceptions, and trust in vaccines [15].

The present study explored multiple factors, such as cultural, social, and structural influences, influencing HPV vaccine decision-making among Cape Verdean immigrant parents. Understanding these factors is critical to developing culturally tailored education and intervention strategies for this population, which has been largely overlooked in HPV research.

## 2. Materials and Methods

### 2.1. Study Design

This qualitative study used a descriptive and exploratory approach [22]. Qualitative methods provide in-depth insights into cultural values, structural barriers, and personal beliefs, especially important for underserved populations with limited prior research [23,24]. The social ecological model (SEM) [25] was adopted to guide the exploration of factors at the intrapersonal, interpersonal, community, organizational, and policy levels that influence HPV vaccination decision-making. Combining this framework with qualitative enriches the development of culturally relevant public health interventions tailored to Cape Verdean families in the U.S. [26,27].

### 2.2. Sample and Participant Recruitment

The study focused on Cape Verdean immigrant parents with at least one child aged 11–17 years. Participants were purposively sampled to ensure variation in age, gender, education, and immigration history [28,29,30].

Eligibility criteria included: (1) self-identified as Cape Verdean; (2) 21 years or older; (3) parent or legal guardian of at least one child aged 11–17; (4) fluent in Cape Verdean Creole, Portuguese, and/or English; (5) willing to provide informed consent for audio recording; and (6) residing in the U.S. for at least 6 months to ensure some experience with the healthcare system. We aimed for a sample size of 40–50 participants, with balanced representation of mothers and fathers to support data saturation [31].

Recruitment took place through Cape Verdean community organizations, health centers, churches, cultural associations, and social media platforms popular among Cape Verdean immigrants. Additionally, multilingual research staff embedded within the Cape Verdean community used community-based and network sampling approaches, including referrals from enrolled participants. These culturally sensitive recruitment strategies were adapted from previous research with immigrant and Afro-diasporic populations [32,33,34].

### 2.3. Data Collection

In-depth, semi-structured interviews were conducted via Zoom [35,36]. The interview guide, informed by the SEM and a review of the literature, covered key domains such as parents’ awareness of HPV and its link to cancer, their knowledge of the HPV vaccine, and attitudes toward its safety, effectiveness, and timing [17,18,33,34]. The guide also examined factors that influence decision-making, including family dynamics, gender norms, cultural values, and fathers’ involvement in health decisions. Participants were asked about their trusted sources of information, such as healthcare providers, community leaders, and peer networks [19,34,37,38,39]. Finally, the guide explored both barriers and facilitators to vaccination, including access to information, experiences with the U.S. healthcare system, language barriers, and concerns related to immigration status [17,18,33,34,37,38,39].

Prior to data collection, participants received a thorough explanation of the study, including their rights and the voluntary nature of participation. Informed verbal consent was then obtained electronically. Afterwards, participants completed an interviewer-administered brief sociodemographic survey via Qualtrics, adapted from previous research with Afro-Caribbean and Lusophone immigrant populations [17,33,34]. The survey gathered data on age, gender, marital status, education level, household income, number of children aged 11–17, primary language spoken at home, and duration of U.S. residence. Acculturation was assessed using a modified version of the Short Acculturation Scale for Hispanics (SASH), tailored to the Cape Verdean context [40], which included measures of language use, media consumption, and cultural identification [40,41].

Following the survey, the qualitative in-depth interview commenced. Interviews lasted approximately 45–60 min and were conducted in either Cape Verdean Creole, Portuguese, or English, based on participants’ preference. Two native, trained Cape Verdean research assistants (M.P. and J.L.), fluent in all three languages, conducted all interviews via Zoom to ensure flexibility and confidentiality. Being Cape Verdean natives familiar with the community’s cultural norms and values, they required minimal cultural sensitivity training. Instead, they received targeted guidance on conducting respectful interviews, upholding ethical standards, and managing emotionally sensitive or distressing responses. Regular team meetings facilitated discussion of challenges and reinforcement of best practices.

To ensure comprehensive coverage of perspectives, a sample size of 40–48 participants was determined to be sufficient [31]. Data saturation was monitored throughout the data collection process and preliminary analysis to guide this determination.

### 2.4. Data Analysis

Data were analyzed using thematic analysis guided by the SEM, which helped identify how factors at the intrapersonal, interpersonal, community, organizational, and policy levels influence parents’ decision-making regarding HPV vaccination for their children [25,42].

All interviews were transcribed verbatim in the original language and anonymized to ensure participant confidentiality. Most interviews were conducted in Cape Verdean Creole and professionally translated into Portuguese for analysis, as two researchers with expertise in qualitative data analysis (DLN and ACL) are native Portuguese speakers.

A combination of inductive and deductive approaches was employed. The inductive process involved open coding to identify emergent concepts from the data, while the deductive approach applied predefined categories based on the SEM framework. The codebook was developed iteratively, informed by both theoretical constructs and emergent findings.

Two native Portuguese-speaking coders, experienced in qualitative research and fluent in both Portuguese and English, independently coded all transcripts to ensure reliability and reduce individual coder bias. Coding discrepancies were reviewed and resolved through discussion until consensus was reached, and the codebook was refined accordingly to improve clarity and consistency. Data saturation was assessed continuously during analysis, with saturation considered achieved when no new themes or significant insights emerged after approximately 40 interviews [43,44]. Although thematic saturation was reached overall, the smaller number of fathers in the sample resulted in less representation of their perspectives within the themes. This limitation was noted to ensure transparency regarding gender representation in the findings.

Themes and subthemes were developed through this iterative process to explore the interaction of multilevel influences on decision-making. The MAXQDA qualitative software was used to manage, code, and organize the data efficiently [45]. Findings were interpreted in relation to the study’s research questions and situated within existing literature. The SEM framework provided a lens through which to contextualize results within the broader sociocultural and policy environments, highlighting the importance of community-specific, and culturally tailored interventions [25].

### 2.5. Ethical Considerations

This study was approved by the Institutional Review Board at the University of Massachusetts Boston (Protocol #3282). All participants gave informed verbal consent, which was documented by a researcher. Participant confidentiality was protected through anonymization of data and secure storage. Participants were reminded of their right to withdraw at any point without penalty.

## 3. Results

### 3.1. Sample Characteristics

Participant demographic characteristics are summarized in Table 1. The sample included 45 Cape Verdean parents (18 fathers and 27 mothers), all of whom were born in Cape Verde. The average age was 42.6 years, with fathers significantly older than mothers (50.0 vs. 41.1 years, *p* = 0.05). Most participants were married or living with a partner (60.0%) and had a high school education (84.4%). Household income was above $50,000 for the majority (66.7%), with no statistically significant gender differences observed across income or educational attainment.

While most parents reported speaking Cape Verdean Creole at home (95.6%) and a majority rated their English proficiency as “very well” or “well” (75.5%), fathers more often had lower SASH acculturation scores (<2.99) than mothers (94.4% vs. 70.4%, *p* = 0.05), suggesting lower acculturation among fathers. Although not statistically significant, trends indicated that mothers had lived in the U.S. longer than fathers and were more likely to have public health insurance.

In terms of healthcare-related outcomes, significant gender-based differences were observed. Mothers were significantly more likely than fathers to report having received a healthcare provider recommendation for the HPV vaccine (*p* = 0.03) and to report that their child had initiated the HPV vaccine series (*p* = 0.03). Nonetheless, overall uptake remained low, with only 13.7% of parents reporting that their child had received at least one dose. These findings suggest important gender differences in parental engagement with healthcare providers and HPV vaccine decision-making, particularly among Cape Verdean fathers, who appear less connected to vaccination guidance and more likely to have lower acculturation levels. However, among parents whose children were unvaccinated (n = 44), both mothers and fathers expressed similarly low intent to vaccinate their child in the next 12 months, with only 11.4% indicating they were likely to do so. 

### 3.2. Themes

The analysis revealed several key themes across the multiple levels of the SEM, presented below. Additional details, including illustrative codes and exemplary quotations, are available in the Supplementary Materials (see Table S1).

#### 3.2.1. Intrapersonal Level: Knowledge, Beliefs, and Misconceptions

##### Theme 1: Limited or Inaccurate Knowledge of HPV

Almost half of the parents (46.7%) expressed little to no knowledge about HPV. Many had never heard of the virus or confused it with other health conditions. For example, several admitted outright unfamiliarity. *“To be honest, I don’t even know what that is”* (Mother #4), *“Honestly, I don’t know much about it”* (Mother #6), *“No, this is my first time hearing about it, so I don’t know anything”* (Mother #13), and *“I’ve heard of it, but I don’t really know what it does or why it’s important”* (Father #2).

Others offered inaccurate descriptions, such as *“The virus could cause a fibroid, and the fibroid could lead to cancer”* (Mother #3) or *“Does it have anything to do with glaucoma?”* (Father #4). Some associated HPV with unrelated diseases like AIDS. *“HPV, it seems like AIDS to me”* (Mother #20). These responses indicate widespread confusion and misinformation at the individual level, which likely hinders informed prevention decisions.

##### Theme 2: Confusion About Transmission

Parents demonstrated varying levels of understanding regarding HPV transmission. While some correctly identified it as a sexually transmitted infection, such as saying, *“It’s one of those sexually transmitted diseases”* (Mother #1) and *“HPV is a virus we get through sexual relations”* (Mother #22), others believed it could also be spread through non-sexual means, including blood transfusions. *“It could be from sex or blood transfusion”* (Mother #16). A similar misunderstanding was echoed by one father. *“I think you can get it from dirty environments or if someone coughs on you”* (Father #17). This lack of clarity reflects broader gaps in health literacy around sexually transmitted infections within the community.

##### Theme 3: Associations with Cervical Cancer and Sexually Transmitted Infections (STIs)

Some parents were aware of the link between HPV and cancer, particularly cervical cancer. Statements like *“HPV, yes, it’s linked to cancer cervical cancer”* (Father #5), “It causes cancer in the cervix” (Mother #12), and *“It’s related to cancer, particularly the cancer that affects the cervix”* (Mother #12) illustrate partial but relevant understanding.

However, the association was often framed in gendered terms, with many assuming only women are at risk. For example, one mother mentioned, *“It’s one of the viruses that cause cancer in the cervix, and men don’t have a cervix”* (Mother #1), and a father noted, *“[…] it affects girls…”* (Father #7). While a few parents noted that both sexes could be affected, the general perception emphasized female vulnerability. One mother said, *“I think girls are at more risk than boys”* (Mother #15).

In addition to the gendered perceptions of cancer risk, some fathers demonstrated a basic awareness of HPV as a sexually transmitted infection. While their understanding varied, several acknowledged the mode of transmission in general terms. For example, one father noted, *“I know it’s one of the most commonly transmitted viruses through sex and also know it can be prevented”* (Father #1). Another remarked, *“[…]* if *I’m not mistaken, it’s that one that’s sexually transmitted?”* (Father #10), revealing some uncertainty yet associating HPV with sexual activity. Similarly, another father stated, *“HPV, I believe is a virus we get through sexual relations”* (Father #18), while yet another simply described it as *“[…] a virus that is sexually transmitted”* (Father #11). These responses indicate that although knowledge among fathers was generally limited and sometimes vague, there was at least a partial recognition of HPV’s sexual transmission, which may serve as a foundation for targeted education and engagement strategies.

This theme elucidates the interplay between intrapersonal beliefs and uncertainty and interpersonal communication dynamics, underscoring how gendered assumptions shape awareness and risk perception. The way parents perceive HPV’s link to cervical cancer and who they believe is at risk is shaped by individual-level knowledge (intrapersonal), which often includes confusion or incomplete understanding, particularly regarding gender vulnerability. For example, many parents assume only women are at risk because men do not have a cervix. This intrapersonal misconception then interacts with communication within families or friends (interpersonal level), where these gendered beliefs get reinforced, challenged, or negotiated during conversations. Such interpersonal dialogues can either clarify or perpetuate misunderstandings about who needs vaccination and why, affecting attitudes toward prevention. In other words, parents’ individual knowledge about HPV risk and transmission is not formed or maintained in isolation; it is shaped and at times transformed by family and social network discussions, particularly those involving culturally rooted gender norms.

##### Theme 4: Preventive Knowledge—Condom Use, Vaccine, and Partner Control

A minority of parents, mostly mothers, identified effective prevention strategies, such as vaccination, condom use, and limiting sexual partners. *“It can be prevented with the vaccine and protection during sex with condoms”,* said one mother (Mother #1). Another elaborated, *“You can also get HPV through anal or oral sex* … *so if there’s any entry point, you need to prevent it”* (Mother #2). Others emphasized trust and communication with sexual partners. *“Make sure you know who you’re with and ask your partner to get tested […]”* (Mother #7). Although these comments reflect a more informed perspective, they were not widely shared among the mothers interviewed.

Among fathers, preventive knowledge related to HPV was generally limited and often expressed with hesitation or uncertainty. One father openly admitted, *“HPV? No, can you explain it to me?”* (Father #2), reflecting a complete lack of awareness. Others demonstrated partial understanding but were unsure of their accuracy. For instance, one stated, *“Maybe by getting the vaccine you can prevent it. But I’m not 100% sure about what I’m saying”* (Father #4). 

A few fathers, however, articulated the purpose of the vaccine with more clarity. As one explained, *“That’s why the HPV vaccine came about, precisely to avoid or minimize the risk of eventually developing cancer and other [problems] …”* (Father #7). Another drew broader connections between HPV prevention and general STI prevention practices. *“Preventing HPV is the same as preventing sexually transmitted diseases, right? It’s about being cautious during sexual activity and preventing it if there’s some form of prevention that we know exists. And well, now the vaccine—like all vaccines—has a role in prevention”* (Father #11). While a few fathers offered informed responses, these were relatively uncommon across the sample, with most exhibiting limited or uncertain knowledge about HPV and its prevention.

##### Theme 5: Lack of Awareness of the Vaccine or Its Purpose

While some parents had heard of the HPV vaccine, most were unaware of its existence, benefits, or intended recipients—especially fathers. For instance, a couple of fathers said, *“I don’t know what that is, HPV what?”* (Father #8) and *“[…] I hadn’t heard about this virus before”* (Father #14). Others expressed vague or partial awareness. *“I know that you can prevent it with a vaccine, right?”*, but added, *“I don’t know much about it”* (Mother #3) or *“I also know there is a vaccine. I don’t know when it is given …”* (Father #10). One mother also noted, *“It’s a vaccine that is not well known … at least within the Cape Verdean community”* (Mother #2), highlighting limited awareness of the HPV vaccine in her community.

A few parents residing in Rhode Island, a state with school-based HPV vaccination, were aware of school-based vaccination programs, especially for girls. *“They gave it to my daughter at school”* (Father #6), and *“It’s a vaccine that’s offered at schools for girls between the ages of 11 and 15”* (Mother #13). Still, many were uncertain about the vaccine’s specifics, such as the eligible age group or whether it applied to boys as well. *“I’m not sure if it’s for both men and women”* (Father #14).

These findings reveal a significant knowledge gap both at the individual (intrapersonal) and community levels, which may hinder HPV vaccine uptake. Many parents lacked essential information about the vaccine’s existence, its purpose, who should receive it, and its benefits. This limited understanding was further compounded by community factors—such as cultural beliefs, social norms, and misinformation—that contribute to widespread uncertainty. Even among parents familiar with school-based vaccination programs, confusion persisted regarding eligibility criteria, gender inclusivity, and the appropriate timing for vaccination. These gaps undermine informed decision-making and reduce the likelihood that parents will vaccinate their children.

##### Theme 6: Hesitancy Due to Uncertainty

Despite some awareness of the vaccine, uncertainty and misinformation—primarily at the intrapersonal level—contributed to parental hesitancy. As one mother explained, *“I heard about the vaccine, but I’m not sure if I would take it. I would need to know more”* (Mother #22). Similarly, a father shared, *“I would be hesitant about vaccinating my children because I don’t know how it (the vaccine) affects people”* (Father #1), highlighting gaps in personal knowledge and perceived risk. Concerns were sometimes influenced by rumors. *“I’ve heard people say they’re afraid of the vaccine. They think it’s harmful”* (Mother #17).

#### 3.2.2. Interpersonal Level: Family, Culture, and Decision-Making

##### Theme 1: Parental Roles and Gender Dynamics

Vaccination decisions were often shaped by traditional parental roles, with mothers frequently taking the lead in matters related to children’s health. Some mothers described themselves as the primary or sole decision-makers. *“[…] I’m always the one who makes the decision […]. I never felt the need to talk to his father about it”* (Mother #6), and *“I’m the one who decides”* (Mother #24). For some, their partner’s limited availability contributed to this dynamic. As one mother shared, *“My husband is a very busy man, so since our children were born, I’ve been the one taking them to the doctor”* (Mother #26). Some fathers echoed this sentiment, acknowledging their partner’s primary role in healthcare decisions: *“Right now, she’s* [mother] *the one taking them to the doctor … she decides, she takes them and follows the doctor’s guidance”* (Father #15). Several fathers noted that while they were often involved in discussions, their role tended to be more consultative than decisive. *“My wife usually makes the final call, but I always ask questions. I want to understand what the vaccine is for and if it’s really needed”* (Father #6).

In many households, vaccination decisions were made jointly between parents. Some emphasized shared responsibility: *“It’s always a decision made between my husband and me. We always follow the same path”* (Mother #22), *“Always, always involved. We do everything together”* (Mother #21), and *“We made the decision together because my daughter’s father, well, he’s a doctor”* (Mother #2). Fathers also described their involvement: *“I inform myself, consult my wife, and then we decide what to do”* (Father #5), and *“That’s a decision between me and my wife. She knows more than I do about medicine. She researches a lot online … we talk about it and weigh the pros and cons”* (Father #10). Even in households where one parent took the lead, dialogue was often maintained: *“Usually, I make these kinds of decisions, but I always talk to their father too … and he always agrees”* (Mother #7), and *“If she says no, I give her my opinion”* (Father #6). Still, some acknowledged that traditional gender expectations shaped these roles: *“Usually, it’s the mother because she’s the one who takes them to appointments …”* (Father #14).

This theme highlights how interpersonal gender dynamics shape the way individual knowledge, beliefs, and hesitations about vaccination are expressed and acted upon within families. These results suggest that mothers, often positioned as primary caregivers, typically lead health-related decisions, making their beliefs about HPV vaccination particularly influential. Fathers tend to play a more consultative or supportive role, and their concerns may carry less weight unless actively discussed. These dynamics reflect broader cultural norms around gender roles and show how intrapersonal beliefs and interpersonal communication intersect to shape vaccination choices.

##### Theme 2: Communication with Children: Involving Youth in HPV Vaccine Decisions

Open communication within some families played a significant role in shaping vaccination decisions. Some parents emphasized the value of including children in vaccine decision-making, particularly as they grew older. As one father explained, *“Of course, they always have the option—especially now that they’re over 10—to know what’s being put into their bodies”* (Father #2). Another noted, *“Since […] is older now—she’s 17—if she says she won’t take it, we can’t force her. We just advise”* (Father #5). Similarly, one mother shared, *“My daughter is like a journalist—anything that goes into her body she needs to understand—so the conversation must come first”* (Mother #21). These parents felt that open discussions about the HPV vaccine fostered understanding and promoted shared decision-making. In such families, older children were often actively involved in health decisions, supporting their growing autonomy.

In contrast, some parents believed that younger children were not mature enough to meaningfully contribute to these decisions and felt it was their responsibility to guide them. As one mother put it, *“I will talk to my daughter, but not yet … she’s too young, she doesn’t really understand. I make the best decision for her now”* (Mother #13). Another added, *“Kids of [this age] can’t really grasp all of it, so it’s up to us to protect them”* (Mother #18).

##### Theme 3: External Influences on Parental Decision-Making: The Role of Social Networks and Healthcare Providers

Beyond the nuclear family, parents reported mixed engagement with friends and extended family. Some did not discuss vaccination with others, believing it was a personal matter. *“Their opinion doesn’t influence mine”* (Mother #7), *“Others’ opinions don’t affect me” (Mother #23),* and *“You shouldn’t let someone influence you on something”* (Mother #14). These results show that some families keep vaccination decisions private, limiting outside influence likely due to cultural taboos and discomfort around HPV that hinder open conversations, sustaining misinformation or hesitation.

Other parents, however, valued conversations with trusted individuals. *“It depends on who. I don’t just listen to anyone. It has to be someone I trust—family or friends whose decisions I respect”* (Father #10), and *“I always talk to them* [family members and trusted friends] *to understand what’s recommended at each stage”* (Father #11), suggesting that for some families, social norms and trust in interpersonal networks mediate vaccine decision-making, connecting community and interpersonal levels.

Health professionals play an especially pivotal role in family communication and decision-making. Many parents cited discussions with doctors as influential. *“I always go by my doctor’s opinion … she explained it would be very useful for boys”* (Mother #8), and *“The doctor said it prevents other diseases, and that he needed to take it, so I gave it”* (Mother #10). Others described gaining awareness through clinical settings or research participation. *“Honestly, I learned about it through you (researcher) and studied it”* (Father #2), and *“My doctor already talked to me about these vaccines”* (Mother #7). Trust in doctors remained high, with statements like *“If the doctor thinks it’s important, I agree”* (Mother #11) and *“If my child’s doctor recommends it, I’ll follow it”* (Father #4) underscoring how interpersonal trust and medical guidance often went hand in hand.

Nonetheless, some fathers noted that their experiences in clinical settings influenced their perspectives—particularly when they felt excluded from discussions. *“Sometimes the doctor just speaks to the mother, like I’m not even there. That makes it harder for me to ask anything”* (Father #2), indicating how healthcare providers at both interpersonal and organizational levels directly support or hinder family-level communication and decision-making.

##### Theme 4: Cultural Beliefs, Taboos, and Misinformation

Parents’ cultural background, especially ties to Cape Verde, influenced their perspectives and decision-making. Some reflected on how past experiences or cultural practices shaped their current approach. *“We talk and decide because we came from Cape Verde, and the decisions you make always carry your past into the future”* (Mother #16). One mother noted, *“Right now, the younger ones, that’s just between me and their mother […]. We decide: ‘yes, we’ll do it”* (Father #5), illustrating how parental authority is culturally normalized in decisions for young children.

Discussions about HPV were often limited by taboos or discomfort related to its status as a sexually transmitted infection. This was not always explicitly stated, but parents indicated that lack of knowledge, generational, or religious differences created additional barriers to open dialogue. *“No, not everything … Because I have my way. Let me explain: all my siblings are Adventist, I’m Catholic. That’s already a big difference—we don’t think alike. So, when it comes to talking about sex and stuff like that, there are differences too”* (Father #1). Another father shared, *“I didn’t know much about HPV at first. I had to do my own reading to feel comfortable, especially since it’s something to do with sex and kids”* (Father #11).

Others expressed a desire to avoid external judgment or interference. *“I don’t share my decision with anyone … I give my opinion if someone asks, but I don’t say whether I’m against it or not”* (Father #15), suggesting underlying concerns about stigma and social expectations, especially within tight-knit or faith-based communities. Some fathers expressed concern about community gossip or stigma, especially around vaccines related to sexual health. *“In our culture, we don’t really talk about those things [STIs]. It’s hard for fathers to ask questions without feeling judged or embarrassed”* (Father #9).

Community rumors and informal networks also shaped perceptions of the vaccine. One mother noted, *“I’ve heard people say they’re afraid of the vaccine. They think it’s harmful”* (Mother #17). Another shared, “Some people say the vaccine can cause infertility, and that scares parents” (Mother #8). A father expressed similar concerns shaped by word of mouth. *“People talk … they say the vaccine is new and could have long-term effects we don’t know about”* (Father #6). These comments illustrate how misinformation circulating within the community contributes to uncertainty and vaccine hesitancy.

Together, these results underscore how cultural norms at the interpersonal level—within families and close social circles, interact with community attitudes and stigma to shape individual beliefs and behaviors. Taboos around sex, religious beliefs, and generational divides often limit open discussions about HPV and vaccination, reinforcing misinformation or fear. These interpersonal and community dynamics can intensify individual uncertainty and mistrust, making vaccine hesitancy not just a personal concern but a socially reinforced one.

#### 3.2.3. Organizational Level: Health System and Institutional Touchpoints

##### Theme 1: Healthcare Providers as Trusted Sources

Healthcare providers, particularly doctors and nurses, were the primary and most trusted sources of HPV vaccine information for many parents. Parents frequently encountered information during routine visits to clinics and hospitals, where they received brochures or verbal explanations or learned about the vaccine through observation and inquiry. One mother shared, *“Talking to my doctor, right? I already know, I learned about it (vaccine) because there’s that exam, we women do every three years if everything is normal, the Pap smear, right?”* (Mother #1). Another stated, *“At school or in the hospital you read pamphlets, in clinics I usually see them”* (Mother #2). Several participants noted that their first exposure to HPV education came from these settings. *“I remember when my youngest … the older one also got it, so when the younger one was old enough, I went to the hospital and they explained what to do, how it works—it’s three doses”* (Mother #15), and *“I saw it on a flyer at the doctor’s office”* (Mother #26).

Some parents expressed appreciation for the U.S. healthcare system, describing it as informative and structured. For instance, one mother emphasized, *“At every child’s doctor appointment, they give you pamphlets with all the vaccine information—even before your kids take them, you can bring them home, read them, do your research, and decide”* (Mother #1). Others valued the step-by-step communication, such as *“When you go to the doctor, they explain it before giving the vaccine … the nurse explains, then gives you that paper to read”* (Mother #5) and *“When we go for a physical exam, there are things the doctor discusses when your child reaches a certain age”* (Mother #16).

However, not all parents had positive experiences. A lack of communication from providers emerged as a significant concern. Some parents felt left out of critical information about the vaccine, with one stating, *“To this day, I haven’t heard anyone talk about it, not even my doctor has said anything”* (Mother #9). Another explained, *“Since it’s something that was never recommended by my doctors … I want to have more information before anything”* (Father #9). Others called for more effective, empathetic communication from medical professionals, emphasizing the importance of informed, not pressured, decision-making. As one participant noted, *“They should inform, communicate it gently, not make it feel mandatory. People should feel free”* (Mother #10). Another added, *“They can explain it, and also explain the benefits, the consequences, the results … what it can do”* (Father #15). These points underscore how the quality of organizational communication affects intrapersonal vaccine attitudes and community trust.

##### Theme 2: Schools, Churches, and Community Organizations as Trusted Channels for Health Communication

Beyond traditional healthcare settings, schools and churches emerged as trusted and culturally resonant venues for health communication. Some parents recalled first learning about HPV through school-based initiatives and viewed schools as important spaces where both children and parents could engage in health education. One mother shared, *“I also learned about HPV at school, when they had planned to give the vaccine to 10-year-old children. My daughter was included, and I got curious to understand why they were giving it”* (Mother #3).

Several others affirmed the potential of schools to act as bridges between children and parents. *“School and church help a lot. Especially school—it’s one of the best places to inform parents”* (Mother #3), and *“Through school, and from school it reaches the parents”* (Father #12). In some cases, school-based health discussions even helped overcome cultural barriers to parent–child conversations. One mother recalled, *“One day, my daughter came home and said, ‘Mommy, the teacher talked to us about sex, do you think that’s right?’ I said, ‘Yes, that’s exactly where you should be learning that’”* (Mother #8).

Churches were also seen as trusted institutions capable of reaching community members in meaningful ways. *“At church it’s always better because people are in a spiritual environment, they’re more attentive,”* said one participant (Father #11), while another emphasized that schools and churches work best because *“parents who take their kids to school, who get them into school, and take them to church, are more open to these things”* (Father #1).

In addition to schools and churches, community-based organizations (CBOs), especially Cape Verdean associations, were noted as effective platforms for outreach. One mother shared, *“Cape Verdean associations putting posters there, doing events, we always have open conversations with women, we gather mothers to talk about this”* (Mother #21). Another recommended broader outreach strategies through trusted voices. *“It could be someone in politics, or a doctor, a social media influencer, or an artist … Since we are a small Cape Verdean community, word spreads fast”* (Father #2). These insights highlight how organizational, and community structures can work together to counter misinformation and support intrapersonal decision-making.

##### Theme 3: Language Barriers and Cultural Accessibility in Healthcare Services

A recurring barrier to effective communication was the lack of HPV-related information in languages other than English. Many parents explained that while materials were available in clinics, they were often inaccessible to non-English speakers. This not only created confusion but also contributed to broader inequities in knowledge and trust. One parent emphasized the need for culturally tailored materials. *“There should also be information about vaccines in Creole: which ones to give, at what ages, how many doses. Everything, like a table in Creole, so people can learn the importance of vaccines”* (Mother #10). Another explained, *“If you don’t speak or read English … how do you get that information for yourself?”* (Father #5).

Despite these strengths, many parents identified language as a major barrier to understanding vaccine information. One mother explained, *“Most information I get from the health clinic is in English, and it’s hard to understand. It would help if they had brochures in Creole or Portuguese”* (Mother #22). A father similarly noted, *“Sometimes the doctor explains things too fast, and if it’s not in Portuguese or Creole, I miss important details. It makes it harder to ask questions or feel confident about the decision”* (Father #5). These accounts reveal that linguistic access remains a key gap, even within a well-resourced system, limiting meaningful engagement in healthcare decisions.

Some parents expressed hope that translating materials into Creole or Portuguese could improve comprehension and trust. *“Yes, or at least in Creole—Creole or Portuguese instead of English”* (Father #8) and *“Having a paper with clear information in Portuguese or Creole”* (Father #10) were common suggestions, highlighting how language is not just a technical issue, but a crosscutting structural barrier that limits access, reinforces dependency, and reduces empowerment in health-related decision-making.

#### 3.2.4. Community Level: Social Norms, Misinformation, and Media Influence

##### Theme 1: Community Norms, Misinformation, and Resistance to HPV Vaccination

Within the Cape Verdean community, cultural norms and generational attitudes contributed to resistance toward HPV vaccination. Some parents described a general sense of distrust or hesitancy rooted in cultural taboos or long-standing beliefs. Vaccination related to sexually transmitted infections was sometimes viewed with suspicion or deemed unnecessary. As one mother noted, *“People in the community say things like, ‘Why should we vaccinate for that? Our kids don’t need it.’ They don’t understand it’s prevention”* (Mother #1). Another added, *“Some people think if you talk about this vaccine, you’re already thinking the child will be sexually active—that’s taboo”* (Mother #3). These views reflect deeper cultural discomfort around adolescent sexuality, which hinders open dialogue and informed decision-making within families and the community.

Compounding these cultural barriers, misinformation circulates widely, especially concerning vaccine safety and necessity. One father commented, *“Some say vaccines are not trustworthy or they cause harm. They just repeat what they heard without checking”* (Father #7). Similarly, a mother noted, *“People in the community say the vaccine isn’t safe, that it can mess with your child’s health, but they don’t really have facts—just what others have told them”* (Mother #5). These social beliefs and community narratives reinforce hesitancy, even when accurate medical information is available.

Parents also pointed to a lack of accessible, culturally relevant education on HPV and the vaccine, limiting informed decision-making. One mother stated, *“I think it’s important to have more information about HPV. If more people knew about it, it would be easier to prevent it”* (Mother #3), while a father observed, *“People don’t talk enough about prevention”* (Father #16). Together, these responses illustrate how community norms, misinformation, and insufficient educational outreach converge to shape HPV vaccine hesitancy in this population.

##### Theme 2: Influence of Media and Social Networks on HPV Vaccine Information

Parents described varied experiences receiving information about HPV and vaccination through media and social networks. Some actively engaged with platforms like Facebook, WhatsApp, and Cape Verdean radio stations, where they learned about the HPV vaccine or discussed health topics with others. As one mother shared, *“I saw something on Facebook about the HPV vaccine and started asking questions”* (Mother #6). WhatsApp groups were frequently mentioned as channels spreading both helpful information and confusing or contradictory messages. *“Sometimes they send vaccine videos or stories, but you don’t know what’s true”* (Father #5), reflecting challenges in discerning reliable information.

Several participants emphasized the value of community-based dialogue as a complement to media outreach. *“We should have community meetings, where mothers can ask questions freely. When you’re comfortable, you learn better”* (Father #4). Another noted, *“We need to create a safe space, maybe a women’s health day or a church event, to talk about these things together”* (Mother #14). These comments highlight a strong desire for trusted, culturally relevant forums that foster open conversations around HPV vaccination.

Cape Verdean radio was also identified as a trusted and culturally appropriate medium. *“Radio is good, especially when they speak Creole, it reaches the older generation too”* (Mother #2), one mother explained. However, many participants noted limited access to or absence of HPV-specific information in local media and community communications. *“We don’t hear much about it here, not on the radio, not on TV. I only knew because of my doctor,”* remarked a father (Father #10). This indicates that despite the potential of media platforms to educate diverse audiences, current HPV messaging remains scarce, representing a missed opportunity to increase awareness and acceptance in the community.

##### Theme 3: Leveraging Community-Based Organizations for Culturally Relevant Education

Recognizing the influence of community norms, many parents suggested leveraging community-based organizations, particularly Cape Verdean organizations, schools, and churches, to promote vaccine awareness. *“If someone in the Cape Verdean Association talks about it, people will listen—it’s one of the best ways to get through”* (Mother #21), one participant suggested. Others highlighted the need for advocacy through local influencers or diaspora leaders. *“It could be a Cape Verdean nurse, someone they know from the community, talking about it in Creole—people would take that seriously”* (Father #6).

Again, churches, schools, and community events were also identified as potential avenues for engaging families in open conversation about HPV and vaccination. One parent shared, *“We should have community meetings, where mothers can ask questions freely. When you’re comfortable, you learn better”* (Father #4). Another parent emphasized, *“We need to create a safe space, maybe a women’s health day or a church event, to talk about these things together”* (Mother #14), underscoring the desire for community-based interventions that are not only informative but also culturally and linguistically aligned with the population they aim to serve.

#### 3.2.5. Policy Level

##### Theme 1: U.S. National Vaccine Program and Parental Experiences

Parents generally expressed appreciation for the comprehensive healthcare system in the U.S., particularly its inclusion of the HPV vaccine as part of routine care. Mothers especially emphasized the value of receiving vaccine-related information during regular pediatric appointments. One mother shared, *“Always, thank God, America has a very advanced healthcare system. And during your kids’ appointments, the doctors always give you pamphlets with all the information about vaccines, even before the kids get them, you can take them home, read them, do your research, and decide if you want to give them to your children”* (Mother #1). Another noted, *“Here, everything is more organized. The doctor explains, they give you papers to read, and you have time to think. That doesn’t happen the same way back home”* (Mother #3). A father echoed, *“They don’t just give the vaccine without telling you. The doctor talks to you, explains why it’s important, and gives you time to ask questions. That helps parents feel more comfortable and involved”* (Father #2).

These positive experiences reflect a general sense of trust in the U.S. healthcare system. According to some parents, particularly those with stronger English proficiency, access to clear, written information about the HPV vaccine enhanced their confidence and ability to make informed decisions for their children.

##### Theme 2: Policy Gaps in Public Health Communication

Although the HPV vaccine is part of routine immunization schedules in the U.S., many Cape Verdean parents reported receiving limited or unclear information about the vaccine’s purpose, benefits, and safety. While some discussions took place during healthcare visits, parents described a lack of consistent, standardized messaging that clearly communicated the vaccine’s link to cancer prevention. One mother noted, *“They give the information but don’t explain. They just say, ‘Here’s the HPV vaccine,’ but they don’t explain anything”* (Mother #3). Another added, *“I kept asking the doctor why this vaccine is needed, but they just said it’s routine. I wanted to know more about how it works, but I never got a clear answer”* (Mother #15).

These communication breakdowns reflect broader policy-level shortcomings—namely, the absence of robust public health infrastructure to deliver accessible, culturally relevant vaccine education. Unlike flu shots or COVID-19 vaccines, which were widely publicized through targeted national campaigns, participants reported little exposure to HPV-related messaging from government agencies, schools, or public health institutions. *“I don’t remember getting any flyers or talks about the HPV vaccine from the school. They only remind about flu shots,”* shared one mother (Mother #17), underscoring the missed opportunities for school-based outreach.

Misinformation and confusion also persisted due to the lack of coordinated public education efforts. As one mother shared, *“The first time I heard about it, I got worried when they said ‘HPV.’ I didn’t know what HPV was. I thought it was because of the coronavirus. I asked, ‘What vaccine is this?’ I got scared. Later I went to find out, I asked, and they told me it’s against a virus that causes cancer”* (Mother #3). Similarly, a father stated, *“No one explained that HPV can cause cancer. I thought it was just another vaccine like the flu shot”* (Father #13). These accounts point to systemic gaps in health education, particularly the lack of multilingual, culturally appropriate materials disseminated at the national or state level. Overall, while healthcare providers are often the first point of contact for vaccine-related information, the absence of centralized, policy-driven efforts to promote HPV vaccination in immigrant communities has left many families underinformed.

##### Theme 3: Structural Access Barriers and Uncertainty Around Vaccine Coverage

In addition to unclear messaging, many parents described policy-level access barriers, particularly related to insurance coverage and public health outreach. Although the HPV vaccine is generally covered under U.S. preventive care guidelines, several participants were unsure whether they would be billed for it. One mother shared, *“I wasn’t sure if the vaccine was covered by our insurance, so I didn’t know if we had to pay for it or not. That made me hesitate”* (Mother #26). These uncertainties were compounded by the absence of centralized communication from insurance providers or health agencies to clarify coverage or promote vaccine awareness.

Others pointed out gaps in school-based outreach, in contrast to widely promoted vaccines like the flu shot. *“I don’t remember getting any flyers or talks about the HPV vaccine from the school. They only remind about flu shots,”* one mother noted (Mother #17). These missed opportunities reflect a broader absence of mandated, culturally tailored, policy-driven campaigns that specifically target immigrant populations.

Overall, these examples point to a policy-level failure to implement consistent, equity-focused strategies that inform families, particularly immigrant and low-English-proficiency households, about vaccine access, costs, and benefits. This contributes to confusion, hesitancy, and delays in uptake, despite the vaccine’s routine inclusion in the national immunization schedule.

## 4. Discussion

This study explored Cape Verdean parents’ knowledge, attitudes, and decision-making processes regarding the HPV vaccine, guided by the SEM [25]. Findings highlight how individual beliefs and attitudes are embedded within broader familial, institutional, cultural, and structural contexts. They also underscore how knowledge gaps, misinformation, and systemic barriers intersect across SEM levels to shape vaccine attitudes and behaviors.

At the individual level, many parents held inaccurate or incomplete beliefs about HPV, often confusing it with unrelated conditions such as fibroids or AIDS. Inadequate knowledge about the HPV vaccine further contributed to confusion, particularly around eligibility, purpose, timing, and the importance of vaccinating boys. Even among those aware of the vaccine, many did not understand its link to various cancers or how HPV is transmitted. These misconceptions were not isolated but reflected broader trends in underserved populations with limited access to culturally appropriate health education [3,4,18,19,34]. These findings are consistent with studies conducted among African diaspora communities, including Somali, Ethiopian, and Nigerian immigrants, where limited HPV knowledge, stigma around sexual health, and concerns about vaccine safety have similarly hindered uptake [34,46,47,48]. In those populations, as in this study, confusion was often reinforced by cultural taboos around discussing sexuality, reinforcing silence at both individual and family levels [18,38].

However, prior research also shows that targeted educational efforts, especially those using native languages and trusted messengers, can significantly improve knowledge and attitudes, even in low-literacy groups [47,49,50,51,52,53,54]. Our findings echo these results, suggesting that culturally relevant health communication can shift vaccine perceptions and support informed decision-making [47].

Social and family networks were powerful influences on vaccine decisions. Mothers were typically the primary decision-makers, although fathers, older children, and extended family members also played important roles in shaping attitudes and choices. These patterns are consistent with studies on African immigrant families that highlight the central role of mothers in managing health decisions shaped by gendered responsibilities and cultural norms [18,51]. At the same time, misinformation within social circles, especially confusion about vaccine purpose, eligibility, and its link to sexuality, was common and often led to hesitancy or delays. This dual role of interpersonal networks as both trusted information sources and channels for misinformation has been documented in other African diaspora populations, including West African communities in the U.S. and Canada [18,51,55,56].

Despite these challenges, many parents expressed openness to involving their children in health decisions as they matured, particularly when information was presented in respectful and age-appropriate ways. Prior studies have similarly highlighted the importance of family-centered, culturally respectful communication that aligns with values such as modesty, child protection, and community trust [18,51,55]. These findings underscore the value of leveraging social networks through culturally grounded, peer-led outreach. Interventions involving parent ambassadors, community leaders, and trusted peer educators, approaches proven effective in other African diaspora contexts, could play a vital role in building vaccine confidence [49,50,51,52,53,54].

Healthcare systems, schools, churches, and community organizations played critical roles in shaping parents’ awareness and access to HPV vaccination. While many parents expressed trust in healthcare providers, they also described inconsistent communication and limited cultural responsiveness. This aligns with previous research showing that a strong, clear provider recommendation is one of the most influential factors in vaccine uptake, particularly when delivered by culturally competent or co-ethnic professionals [18,34,46,53].

However, language barriers and limited availability of Cape Verdean Creole materials significantly impeded understanding. These challenges mirror those identified in other studies of African immigrants, where limited English proficiency, cultural misunderstandings, and unfamiliarity with U.S. healthcare systems constrained access to preventive care [15,21,57,58,59].

Churches and schools were viewed as highly trusted institutions and effective venues for community health messaging. Prior research among African diaspora communities has documented the success of engaging faith-based organizations, immigrant-serving nonprofits, and schools as culturally appropriate outreach hubs [49,50,51,52,53,54,55,60,61,62,63,64]. Participants in this study echoed these sentiments, recommending that HPV-related education be delivered in partnership with schools and churches to enhance credibility and reach. These findings point to the need for organizational partnerships and cross-sector collaboration to ensure consistent, culturally responsive messaging. In line with prior research, provider training, multilingual educational materials, and outreach through trusted institutions are critical strategies to improve vaccine access and trust [50,59,60,61,62,63,64].

At the community level, cultural norms, social stigma, and misinformation played major roles in shaping attitudes toward HPV vaccination. Taboos around sexuality and a broader discomfort with discussing adolescent sexual health fueled skepticism and silence, echoing themes reported in studies of East and West African immigrant groups [47,48].

Digital and social media platforms such as WhatsApp, alongside traditional media like Cape Verdean radio, served as both helpful tools and conduits for misinformation. This is consistent with research on African immigrant communities where digital networks can rapidly spread both culturally resonant health messaging and rumors [48,51]. Parents in this study called for accessible, culturally appropriate education in Cape Verdean Creole delivered through trusted community platforms and messengers.

Community-based organizations, while trusted, were not actively engaged in HPV vaccine outreach. This represents a missed opportunity, especially given their potential to act as cultural and linguistic bridges. Findings from other African diaspora contexts have demonstrated the effectiveness of community-based organizations in vaccine campaigns when given appropriate training and support [46,47,48,57,58,65,66]. Creative community-led approaches, such as culturally relevant videos, comics, and youth-led media campaigns, have proven successful in similar populations (e.g., the #HPVVaxTalks campaign), such as sub-Saharan African immigrants [46]. Co-developing such tools with Cape Verdean youth and families may foster greater engagement and understanding [46,47,48].

At the policy level, parents supported the inclusion of the HPV vaccine in routine adolescent care but noted that public health communication was often fragmented, confusing, and not culturally or linguistically tailored. While access to the vaccine existed, true engagement remained limited due to inconsistent messaging and lack of outreach in Cape Verdean Creole. These challenges reflect findings from broader research showing that immigrant communities require not only physical access to vaccines but also targeted education that is linguistically appropriate and culturally resonant [3,4,14,66]. Multilevel interventions that pair structural access with community-driven education have been successful in reducing disparities in other African diaspora communities and may serve as a model for Cape Verdean communities, considering immigration status, healthcare access, and diaspora-specific histories and needs [65,66].

Moreover, parents in this study emphasized that policy solutions must go beyond improving access to include culturally tailored communication, language equity, and protections that alleviate immigration-related fears. Prior research similarly underscores the need for public health policies that embed equity and education at their core, especially for immigrant and refugee populations [3,4,18,66,67,68].

### 4.1. Implications and Recommendations

Study findings highlight the critical need for multilevel, community-engaged, and culturally and linguistically tailored public health strategies to address vaccine hesitancy and improve HPV vaccine uptake among Cape Verdean communities. Such approaches are essential for building trust, enhancing understanding, and promoting health equity. Suggestions for these strategies are outlined below:

- Develop and distribute culturally and linguistically appropriate HPV education materials in Cape Verdean Creole and Portuguese, using culturally resonant messaging, visuals, and examples to explain HPV, its risks, and the benefits of vaccination for both boys and girls.
- Train healthcare providers and staff in Cape Verdean-specific cultural competence, emphasizing respectful communication, strong vaccine recommendations, and attention to language barriers during clinical interactions.
- Partner with trusted community institutions—such as churches, schools, and Cape Verdean associations—to host vaccine education sessions, mobile clinics, and outreach events that normalize HPV vaccination and encourage family involvement in health decisions.
- Leverage ethnic media and digital platforms, including Cape Verdean radio, community WhatsApp groups, and social media, to counter misinformation and share accurate, accessible vaccine information delivered by trusted local voices.
- Strengthen the role of CBOs by providing funding and support to enable them to serve as intermediaries between public health systems and Cape Verdean families, offering navigation support, education, and culturally relevant resources.
- Advocate for state-level incentives and funding to expand school-based HPV vaccination programs, including requirements or encouragement for schools to actively promote vaccination through accessible clinics and educational initiatives.
- Allocate public health funding to develop and disseminate HPV educational materials specifically in Cape Verdean Creole, ensuring these resources are easily available in healthcare settings, schools, and community centers.
- Implement mandatory cultural competency training programs for healthcare providers that focus on the specific needs, language preferences, and cultural contexts of Cape Verdean families to improve communication and vaccine uptake.

### 4.2. Limitations

The interpretation of this study’s findings should consider some limitations. First, the use of purposive and snowball sampling methods may have introduced selection bias, potentially skewing the sample toward participants who were more engaged or held particularly strong views, thus limiting the range of perspectives represented [22]. Furthermore, the context-specific nature of this research may restrict the transferability of results to other immigrant populations, given the influence of differing cultural backgrounds, socioeconomic conditions, and healthcare access [22].

Although the study included both mothers and fathers, mothers were more numerous and more vocal in interviews. This gender imbalance may reflect caregiving norms within the community and could have influenced the findings, with mothers’ perspectives being more prominent in the analysis.

Self-reported data may also have been subject to social desirability bias, with participants possibly tailoring their responses to align with perceived expectations [69]. While thematic analysis enabled the identification of key patterns across interviews, its interpretive nature introduces the potential for researcher bias, which may have shaped how themes were developed and understood [23,42,43].

Finally, data collection via Zoom interviews may have introduced certain limitations. Some participants could have faced technological barriers, such as unstable internet connections or limited familiarity with the platform, which might have impacted their ability to participate fully. Additionally, the absence of in-person interaction may have constrained the depth of rapport-building and nonverbal communication, potentially affecting the richness of responses. These factors should be considered when interpreting the findings.

Nonetheless, the study offers several notable strengths. The use of semi-structured interviews provided rich, nuanced insights into participants’ experiences and attitudes surrounding HPV vaccination. The sampling approach, although not without limitations, facilitated the inclusion of a demographically diverse group, particularly immigrant fathers, a population frequently underrepresented in public health discourse. In doing so, this study contributes valuable, culturally contextualized evidence to guide future efforts aimed at enhancing HPV vaccine uptake.

## 5. Conclusions

This study revealed that HPV vaccine hesitancy among Cape Verdean parents arises from a complex interplay of individual knowledge gaps and cultural taboos, family dynamics, inconsistent and culturally unresponsive communication from healthcare and community organizations, and broader social stigma and misinformation. Language barriers and limited availability of educational materials in Cape Verdean Creole further impede understanding and access, while immigration-related fears and fragmented public health messaging exacerbate these challenges at the systemic level. Together, these factors highlight the necessity of coordinated, culturally and linguistically tailored strategies that engage families, leverage trusted community institutions, and empower community organizations. Strengthening provider communication through cultural competence training, partnering with faith-based and educational institutions, expanding multilingual outreach through ethnic media, and addressing structural inequities through policy reforms are critical to building vaccine confidence and promoting health equity. Without integrated, community-centered approaches that address these intertwined influences, disparities in HPV vaccine uptake and broader health outcomes will likely persist in Cape Verdean and other underrepresented immigrant communities.

## Figures and Tables

**Table 1:** Sociodemographic and cultural characteristics of the sample (N = 45).

Variables	Total N = 45 (%)	Fathers n = 18 (%)	Mothers n = 27 (%)	*p*-Value
Age (Mean, SD)	42.6 (7.9)	50 (9.1)	41.1 (6.9)	0.05
Marital status				
Married/Living with Partner	27 (60.0%)	11 (61.1%)	16 (59.3%)	0.90
Divorced/Separated/Single	18 (40.0%)	7 (38.9%)	11 (40.7%)	
Educational attainment				
Less than high school diploma	7 (15.6%)	2 (11.1%)	5 (18.5%)	0.51
More than high school	38 (22.2%)	16 (22.2%)	22 (22.2%)	
Household annual income				
<US $50,000	15 (33.3%)	4 (22.2%)	11 (40.7%)	0.20
≥US $50,000	30 (66.7%)	14 (77.8%)	16 (59.3%)	
Religious affiliation				
Catholic	34 (75.6%)	13 (72.2%)	21 (77.8%)	0.67
Other	11 (24.4%)	5 (27.8%)	6 (12.2%)	
Country of birth				
Cape Verde	45 (100%)	18 (100%)	27 (100%)	-
Years living in the U.S.				
<10 years	11 (24.4%)	2 (11.1%)	9 (33.3%)	0.09
≥10 years	34 (40.0%)	16 (38.9%)	18 (66.7%)	
Primary language spoken at home				
Cape Verdean Creole	43 (95.6%)	17 (94.4%)	26 (96.3%)	1.00
English	2 (4.4%)	1 (5.6%)	1 (3.7%)	
How well parent speaks English				
Very Well/Well	34 (75.5%)	15 (83.3%)	19 (70.4%)	0.32
Not Well/Not at All	11 (24.5%)	3 (16.7%)	8 (29.6%)	
SASH score				
<2.99	36 (80.0%)	17 (94.4%)	19 (70.4%)	0.05
≥2.99	9 (20.0%)	1 (5.6%)	8 (29.6%)	
Health insurance				
Public/Government-sponsored	22 (48.9%)	6 (33.3%)	16 (59.3%)	0.09
Private	23 (51.1%)	12 (66.7%)	11 (40.7%)	
Number of children between 11–17 years (N = 51)				
1	41 (80.4%)	17 (94.4%)	24 (85.7%)	0.53
2	10 (19.6%)	1 (5.6%)	4 (14.3%)	
Child gender (N = 51)				
Female	30 (58.8%)	10 (52.6%)	20 (62.5%)	0.20
Male	21 (41.2%)	9 (47.4%)	12 (37.5%)	
Healthcare provider recommended HPV vaccine (N = 51)				
Yes	7 (13.7%)	0	7 (21.9%)	0.03
No	44 (86.3%	19 (100%)	25 (78.1%)	
Child received at least one dose HPV vaccine (N = 51)				
Yes	7 (13.7%)	0	7 (21.9%)	0.03
No	44 (86.3%	19 (100%)	25 (78.1%)	
Likelihood child receiving HPV vaccine in next 12 months (n = 44)				
Extremely/Very Likely/Likely	5 (11.4%)	2 (10.5%)	3 (12.0%)	0.88
Not Too Likely/Don’t Know	39 (88.6%)	17 (89.5%)	22 (88.0%)	

## Data Availability

Due to privacy or ethical restrictions, the data that support the findings of this analysis are not publicly available; however, they are available upon request from the corresponding author.

## Supplementary Materials

The following supporting information can be downloaded at: https://www.mdpi.com/article/10.3390/vaccines13070713/s1, Table S1: Key Themes, Illustrative Codes, and Exemplary Quotations by Levels of the Social Ecological Model (SEM).

## Abbreviations

The following abbreviations are used in this manuscript:

CBO	Community-Based Organizations
CDC	Centers for Disease Control and Prevention
HPV	Human Papillomavirus
SASH	Short Acculturation Scale for Hispanics
U.S.	U.S.
