# Supplementary material for: Social Ecological Influences on HPV Vaccination Among Cape Verdean Immigrants in the U. S.: A Qualitative Study
Source: Vaccines (Basel). 2025 Jun 30;13(7):713. doi: 10.3390/vaccines13070713 (PMC12300976; doi:10.3390/vaccines13070713)
Supplement: Supplementary file 1 [file vaccines-13-00713-s001.zip › vaccines-3678427-supplementary.pdf]

Table S1. Key Themes, Illustrative Codes, and Exemplary Quotations by Levels of the Social Ecological Model (SEM).

Level	Themes	Illustrative Codes	Example Quotations
**Intrapersonal**	**Theme 1:** Limited or Inaccurate Knowledge of HPV	Misconceptions about HPV; Incomplete understanding	"The virus... could cause a fibroid, and the fibroid could lead to cancer."<br>"Honestly, I don't know much about it."<br>"HPV, it seems like AIDS to me."
	**Theme 2:** Confusion About Transmission	Unclear transmission routes; Uncertainty about spread	"It's one of those sexually transmitted diseases."<br>"HPV... is a virus we get through sexual relations…"<br>"It could be from sex or blood transfusion."
	**Theme 3:** Associations with Cervical Cancer and Sexually Transmitted Infections (STIs)	Linking HPV to cancer; Fear of serious outcomes	"HPV, yes, it's linked to cancer... cervical cancer."<br>"It's related to cancer, particularly the cancer that affects the cervix."<br>"It's one of the viruses that cause cancer in the cervix, and men don't have a cervix."
	**Theme 4:** Preventive Knowledge – Condom Use, Vaccine, Partner Control	Partial knowledge of prevention methods; Vaccine awareness	"Using condoms helps, but the vaccine is still new to me."<br>It can be prevented with the vaccine and protection during sex with condoms."<br>"Preventing HPV is the same as preventing sexually transmitted diseases, right? It's about being cautious during sexual activity, in contact, and preventing it if there's some form of prevention that we know exists. And well, now the vaccine—like all vaccines—has a role in prevention."
	**Theme 5:** Lack of Awareness of the Vaccine or Its Purpose	Unawareness of vaccine existence or role	"I didn't even know there was a vaccine for HPV."<br>"I also know there is a vaccine. I don't know when it is given."

Table S1. Key Themes, Illustrative Codes, and Exemplary Quotations by Levels of the Social Ecological Model (SEM).

Level	Themes	Illustrative Codes	Example Quotations
			"I'm not sure if it's for both men and women."
	**Theme 6:** Hesitancy Due to Uncertainty	Safety concerns; Doubts about vaccine efficacy	"I'm not sure if the vaccine is safe for young girls."<br>"I'm not sure if it's for both men and women."
Interpersonal	**Theme 1:** Parental Roles and Gender Dynamics	Parental influence; Gendered vaccine perceptions	"My wife usually makes the final call, but I always ask questions. I want to understand what the vaccine is for and if it's really needed."<br>"I am not sure boys need this vaccine."<br>"No, I'm always the one who makes the decision [...] I never felt the need to talk to his father about it."
	**Theme 2:** Communication with Children: Involving Youth in HPV Vaccine Decisions	Family silence on sexual health; Lack of open discussion; Children involvement in some families	"We don't really discuss sexual health at home."<br>"Kids of 9, 10, 11 years old can't decide these things. They don't have decision-making power."<br>"I talk to her about vaccines... we've discussed why the vaccine is needed."<br>"Of course, they always have the option—especially now that they're over 10—to know what's being put into their bodies"
	**Theme 3:** External Influences on Parental Decision-Making: The Role of Social Networks and Healthcare Providers	Cultural communication norms; Healthcare providers' influence on vaccination; Barriers in clinical communication	"It depends on who. I don't just listen to anyone... it has to be someone I trust—family or friends whose decisions I respect"<br>"I always go by my doctor's opinion... she explained it would be very useful for boys"

Table S1. Key Themes, Illustrative Codes, and Exemplary Quotations by Levels of the Social Ecological Model (SEM).

Level	Themes	Illustrative Codes	Example Quotations
			“Sometimes the doctor just speaks to the mother, like I’m not even there. That makes it harder for me to ask anything.”
	**Theme 4:** Cultural Beliefs, Taboos and Misinformation	Cultural taboos; Myths and misinformation	"In our community, talking about HPV is taboo because the virus is sexually transmitted."<br>“In our culture, we don’t really talk about those things [STIs]. It’s hard for fathers to ask questions without feeling judged or embarrassed.”<br>“I didn’t know much about HPV at first. I had to do my own reading to feel comfortable, especially since it’s something to do with sex and kids.”
	**Theme 1:** Health Care Providers as Trusted Sources	Trust in healthcare providers; Provider recommendations	"My doctor explained the vaccine, and I trust her advice."<br>“I remember when my youngest... the older one also got it, so when the younger one was old enough, I went to the hospital and they explained what to do, how it works—it’s three doses.”
**Organizational**	**Theme 2:** Schools, Churches, and Community Organizations as Trusted Channels for Health Communication	Institutional outreach; Education programs	"My daughter’s school held sessions about HPV vaccination."<br>“I also learned about HPV at school, when they had planned to give the vaccine to 10-year-old children. My daughter was included, and I got curious to understand why they were giving it.”
	**Theme 3:** Language Barriers and Cultural Accessibility in Healthcare Services	Language difficulties; Communication challenges	"The clinic staff spoke mostly English, which is hard for some parents."

Table S1. Key Themes, Illustrative Codes, and Exemplary Quotations by Levels of the Social Ecological Model (SEM).

Level	Themes	Illustrative Codes	Example Quotations
**Community**	**Theme 1:** Community Norms, Misinformation, and Resistance to HPV Vaccination	Social stigma; Vaccine resistance	"If you don't speak or read English, how do you get that information for yourself?"<br>"Some people in our community think vaccines encourage promiscuity."<br>"Some people think if you talk about this vaccine, you're already thinking the child will be sexually active—that's taboo."
	**Theme 2:** Influence of Media and Social Networks on HPV Vaccine Information	Influence of social media; Spread of rumors	"I heard from Facebook that the vaccine causes problems."<br>"Some say vaccines are not trustworthy or they cause harm. They just repeat what they heard without checking."
	**Theme 3:** Leveraging Community-Based Organizations for Culturally Relevant Education	Potential outreach sites; Community programs	"Church groups could be a good place for education."<br>"We need to create a safe space—maybe a women's health day, or a church event—to talk about these things together."<br>"We should have community meetings, where mothers can ask questions freely. When you're comfortable, you learn better."
**Policy**	**Theme 1:** U.S. National Vaccine Program and Parental Experiences	Government programs; Parental attitudes	"…the vaccine program (in Cape Verde) feels pushed on us without explanation."<br>"Here, everything is more organized. The doctor explains, they give you papers to read, and you have time to think. That doesn't happen the same way back home"
	**Theme 2:** Policy Gaps in Public Health Communication	Lack of accessible information; Language barriers	"There isn't enough information in Cape Verdean Creole about HPV vaccines."

Table S1. Key Themes, Illustrative Codes, and Exemplary Quotations by Levels of the Social Ecological Model (SEM).

Level	Themes	Illustrative Codes	Example Quotations
			"I don't remember getting any flyers or talks about the HPV vaccine from the school. They only remind about flu shots."<br>"Sometimes the doctor explains things too fast, and if it's not in Portuguese or Creole, I miss important details. It makes it harder to ask questions or feel confident about the decision."
	**Theme 3:** Structural Access Barriers and Uncertainty Around Vaccine Coverage	Insurance confusion; Lack of school-based outreach	"I wasn't sure if the vaccine was covered by our insurance, so I didn't know if we had to pay for it or not. That made me hesitate."<br>"I don't remember getting any flyers or talks about the HPV vaccine from the school. They only remind about flu shots."
